# Health Care Professionals’ Views of Barriers and Facilitators for Implementing a Fall Risk Screening Tool in Clinical and Public Health Settings

**DOI:** 10.1093/ptj/pzaf018

**Published:** 2025-02-18

**Authors:** Nathalie Frisendahl, Patrik Karlsson, Christina Sandlund, Stina Ek, Erika Franzén, Anne-Marie Boström, Anna-Karin Welmer

**Affiliations:** Division of Physiotherapy, Department of Neurobiology Care Sciences and Society, Karolinska Institutet, 14152 Huddinge, Sweden; Aging Research Center, Department of Neurobiology, Care Sciences and Society, Karolinska Institutet, 17165 Solna, Sweden; Division of Physiotherapy, Department of Neurobiology Care Sciences and Society, Karolinska Institutet, 14152 Huddinge, Sweden; Division of Physiotherapy, Department of Neurobiology Care Sciences and Society, Karolinska Institutet, 14152 Huddinge, Sweden; Division of Family Medicine and Primary Care, Department of Neurobiology, Care Sciences and Society, Karolinska Institutet, 14152 Huddinge, Sweden; Academic Primary Health Care Centre, Region Stockholm, 11365 Stockholm, Sweden; Unit of Epidemiology, Institute of Environmental Medicine, Karolinska Institutet, Nobelsväg 12A, 17165 Solna, Sweden; Division of Physiotherapy, Department of Neurobiology Care Sciences and Society, Karolinska Institutet, 14152 Huddinge, Sweden; Women’s Health and Allied Health Professionals Theme, Medical Unit Occupational Therapy & Physiotherapy, Karolinska University Hospital, 17164 Stockholm, Sweden; Research and Development Unit, Stockholms Sjukhem, 11219 Stockholm, Sweden; Research and Development Unit, Stockholms Sjukhem, 11219 Stockholm, Sweden; Division of Nursing, Department of Neurobiology, Care Science and Society, Karolinska Institutet, 14152 Huddinge, Sweden; Theme Inflammation and Aging, Medical Unit Aging, Karolinska University Hospital, 14157 Huddinge, Sweden; Division of Physiotherapy, Department of Neurobiology Care Sciences and Society, Karolinska Institutet, 14152 Huddinge, Sweden; Aging Research Center, Department of Neurobiology, Care Sciences and Society, Karolinska Institutet, 17165 Solna, Sweden; Women’s Health and Allied Health Professionals Theme, Medical Unit Medical Psychology, Karolinska University Hospital, 17164 Stockholm, Sweden

**Keywords:** Health Care Professionals, Implementation, Injurious Falls, Primary Health Care, Thematic Analysis

## Abstract

**Objective:**

The experiences of health care professionals using new screening tools in clinical and public health settings are crucial to the implementation process. However, further research is needed on their experiences with fall risk screening. This study utilized the integrated-Promoting Action on Research Implementation in Health Services (i-PARIHS) framework to explore health care professionals’ experiences with the First-time Injurious Falls (FIF) screening tool, aiming to identify barriers and facilitators for implementing the FIF tool in primary health care and public health settings.

**Methods:**

A qualitative study with 4 focus group interviews and 7 individual interviews was carried out, using a semistructured interview guide. The interviews were recorded, transcribed verbatim, and analyzed with reflexive thematic analysis. The study included 20 participants (13 females and 7 males), with a mean age of 39 years (range 24 to 54). The participants were working in the primary health care setting (8 physical therapists, 3 occupational therapists, 3 managers, 2 registered nurses, and 1 dietician) and in a public health project (1 physical therapist and 2 health educators).

**Results:**

The analysis resulted in 3 themes: “a valuable tool in clinical practice,” “how to get everyone onboard when implementing fall risk screening,” and “applicable in many areas of health services but not in all” with 4 related subthemes “quick and easy to use for all health care professionals,” “simplifies assessment and creates a platform for discussion,” “need for clear instructions and action list,” and “should be incorporated into daily routines.”

**Conclusion:**

The FIF tool was well-received by participants in practice as it was user-friendly and potentially effective in preventing falls. However, there is room for improvement, particularly in clarifying instructions to mitigate possible misinterpretations. The participants emphasized that implementation of a new screening tool requires favorable organizational conditions such as managerial support, that the tool is easily accessible, and the results are easy to document.

**Impact:**

The FIF tool seems to be a valuable screening tool for predicting first-time injurious falls in older adults, suitable for use by various health care professionals.

## INTRODUCTION

More than one third of the community-living older adults fall each year, and fall-related injuries amongst the older population is a major public health problem. Falls contribute to 5% to 10% of all emergency department visits among older adults, and lead to serious consequences such as longstanding pain, disability, institutionalization, and increased mortality.^1^

Since a previous fall is the most prominent risk factor for subsequent falls, prevention of the first fall has important effects on public health as well as on the daily lives of older adults.^2^ The First-time Injurious Fall (FIF) tool,^3^ was developed for screening first-time injurious falls in community-living older adults. It consists of 3 self-reported questions (age, cohabitation status and Instrumental Activities of Daily Living (IADL) dependency) and 1 balance test (1-leg stance) (Suppl. Material 1). The FIF tool has been validated in 2 cohorts in addition to the development cohort and has shown high predictive values for first-time falls (Harrell’s C statistic ≥0.75 for both females and males) over a 5 years of follow-up period.^4^

It has been suggested that there is a gap between evidence-based knowledge and practice in falls prevention.^5^^,^^6^ One important aspect in the implementation process is to investigate how the FIF tool is perceived by different health care professionals in clinical and public health settings.^7^ To succeed in the implementation process, even if a preventive strategy includes a relatively simple intervention or screening, its interaction with its context (eg, primary health care) may still be highly complex.^8^ Implementation research address barriers and facilitators in different contexts (ie, organization, community, policy/society) to implement fall prevention interventions in clinical settings.^9^ It is important to identify barriers at the pre-implementation stage so they can be addressed early.

Multiple barriers to implementation have been suggested as for example insufficient time for health care professionals to address fall prevention in the context of competing demands and a focus on diagnosis and treatment of specific diseases.^10^ A qualitative study emphasized the importance of providing the health care professionals with support and preparation on how to use a new tool with their patients before the test period begins.^11^ In another study it was suggested that several domains of importance for implementation, for example, knowledge, training and skills may not be captured by quantitative surveys to assess health professionals’ experience of integrated care.^12^ Other studies used qualitative design to explore health care professionals’ experiences to improve care coordination,^13^ strengthening the primary health care,^14^ or the rehabilitation practices for people with stroke.^15^ There lies a need to base the research on patients’ and health care professionals’ priorities and preferences to conduct research with as high quality as possible.^16^

For this study, we utilized the integrated Promoting Action on Research Implementation in Health Services (i-PARIHS) framework.^17^ The i-PARIHS framework offers a systematic approach for identifying potential barriers and facilitators that may impact implementation outcomes. It consists of 4 core elements: the innovation to be implemented, the recipients of the intervention, the context into which the research is to be placed, and the method by which the process is facilitated. The explicit focus on sourcing and utilizing available research evidence is a central construct in the i-PARIHS framework and is done to inform the innovation.

The framework identifies recipients as those individuals who are affected by and can influence the implementation, both at the individual and collective team levels. To enhance our understanding of the recipients within the i-PARIHS framework, we recently investigated patients’ experiences using the FIF tool in primary health care. They described screening for fall risk as meaningful and important for falls prevention.^18^ There is a need to further incorporate the health care professionals’ perspective into clinical practice guidelines and related health resources, since this, as of yet, has not been done on a consistent level, according to Montero-Odasso et al.^19^

The purpose of the present study was to explore health care professionals’ experiences in using the FIF tool to gain insights into the barriers and facilitators for implementing the FIF tool in primary health care and public health settings.

## METHOD

### Design

Epistemologically, we assume that knowledge is subjective and constructed by social realities. Social reality is constructed through phenomena created by those living in a specific social context.^20^ Based on this theoretical framework, a qualitative reflexive thematic analysis using inductive approach was applied in this study,^21^^,^^22^ using focus group methodology (ie, when groups of people are interviewed collectively about a specific topic),^23^^,^^24^ and individual interviews. The rationale for interviewing different groups of health care professionals was to capture perspectives from different professionals that may have different experiences from using the FIF tool. The focus for both kind of interviews were the subjective differences, each individual’s truth describes the social context and reality.^20^ Furthermore, the focus group methodology was considered not be useful for all participants since not all of the participants had used the FIF tool and did not share common experiences.^23–25^

### Settings and Participants

**Figure 1:**
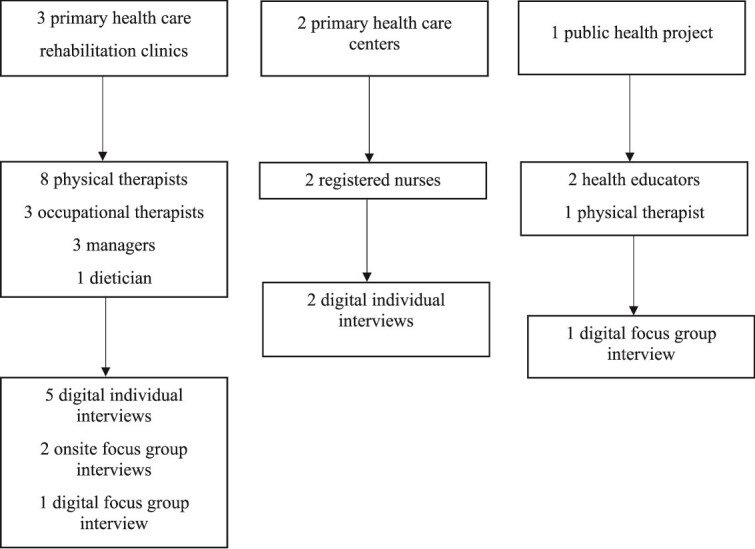
Flow Chart.

Participants were recruited through 3 primary health care rehabilitations clinics, 2 primary health care centers and 1 public health project in Sweden. All participants were working as a health care professional in primary health care or in a public health project. They were informed about the FIF tool by an oral/digital presentation or/and written instruction. The participants who were actively involved in clinical work were asked to use the FIF tool on suitable patients during at least 1 month. A purposive sampling method was applied in this study.^26^ All participants received oral and written information from the main author, outlining the study and informing them that they might be invited to participate in either a focus group or an individual interview. The type of interview depended largely on their position at their workplace, whether they were managers or part of a larger team (Figure 1). All participants who agreed to be included in the study were scheduled for an interview at their workplace or digitally via Microsoft Teams (Microsoft Corp, Redmond, WA, USA), according to their preference.

Background information was collected regarding, sex, age, profession and working place, presented in Table 1. A total of 20 health care professionals (9 physical therapists, 3 occupational therapists, 3 managers, 2 nurses, 2 health educators, and 1 dietician), 13 females, and 7 males agreed to participate in the study.

**Table 1:** Descriptions of Participants

**Sex**	**Age**	**Profession**	**Workplace**
Female	35	Physical therapist	Public health project
Female	28	Health educator	Public health project
Male	32	Health educator	Public health project
Male	33	Occupational therapist	Primary health care
Female	50	Physical therapist	Primary health care
Male	35	Physical therapist	Primary health care
Male	37	Physical therapist	Primary health care
Male	42	Physical therapist	Primary health care
Female	28	Physical therapist	Primary health care
Female	35	Physical therapist	Primary health care
Male	49	Manager	Primary health care
Female	46	Dietician	Primary health care
Female	48	Registered nurse	Primary health care
Female	42	Registered nurse	Primary health care
Female	32	Occupational therapist	Primary health care
Female	47	Manager	Primary health care
Female	44	Manager	Primary health care
Female	47	Occupational therapist	Primary health care
Female	24	Physical therapist	Primary health care
Female	54	Physical therapist	Primary health care

### Data Collection

The data was collected through 4 focus group interviews and 7 individual interviews conducted from June 2022 to January 2023. The number of participants at the various interview sessions varied from 1 to 4 depending on the type of interview. Two of the focus group interviews took place at primary health care rehabilitation clinics. All other interviews were held digitally. Three focus group interviews were conducted by the first author and the second author, with 1 serving as moderator and the other as an observer. The observer took notes about things that could not be captured on the recordings, such as the interaction between the respondents. The rest of the interviews had only the first author as a moderator, without any observer. The interviews were conducted based on semistructured interview guides (Suppl. Material S2).^25^ The interview guides contained several open-ended questions regarding the health care professionals’ experiences from using the FIF tool in primary health care and public health, and barriers and facilitators for implementing the FIF tool. The opening question was “What are your thoughts about injurious falls?” Follow-up questions were then asked to delve deeper into the answers until the questions were exhausted. The time for the interviews varied between 20 and 52 min.

### Data Analysis

**Figure 2:**
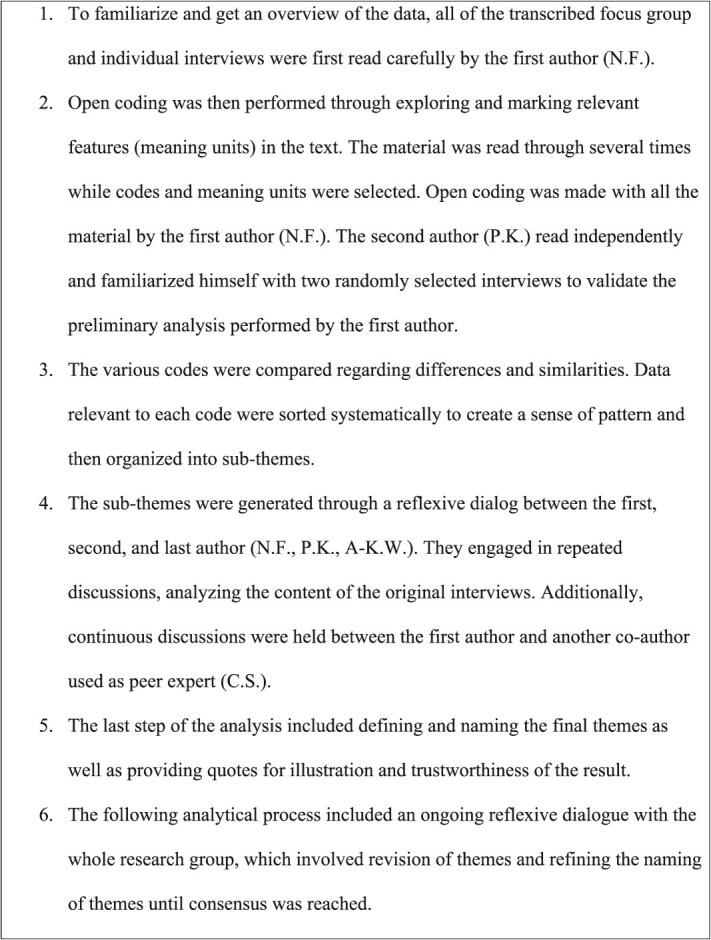
Steps in the Data Analysis.

There was a discussion between the moderator and the observer of what was perceived during the interviews before continuing with the transcription as a first step in the analysis process. The recordings of all interviews were transcribed verbatim by the first author. A qualitative reflexive thematic analysis with an inductive approach was used,^21^^,^^22^ and this method was chosen in order to achieve a broad picture of the participants’ experience. Initially, all the transcribed text was read by the first and the second author, to enable them to familiarize themselves with the material. The analysis then followed 6 phases: familiarization with data, generating initial codes, searching for themes, reviewing themes, defining and naming themes, and producing the report as shown in (Table 2).^21^^,^^22^ To ensure trustworthiness we applied the criteria of credibility, dependability, confirmability, and transferability.^27^ One strength in terms of credibility was the detailed description of the analytical process (Figure 2), and that, in addition to the first author, 2 other researchers (P.K., A-K.W.) were involved in analyzing the data. Additionally, 1 co-author (C.S.) acted as a peer expert to verify that the findings reflected the content of the interviews. All authors were experienced in qualitative research approaches and knowledgeable in different qualitative methods.

**Table 2:** Data Analysis Process From Code to Theme*^a^*

**Theme**	**Subtheme**	**Code**
A valuable tool in clinical practice	Quick and easy to use for all health care professionals	Short test
A valuable tool in clinical practice	Quick and easy to use for all health care professionals	Comprehensible questions
A valuable tool in clinical practice	Simplifies assessment and creates a platform for discussion	Captures what’s needed
A valuable tool in clinical practice	Simplifies assessment and creates a platform for discussion	Creates an overview
How to get everyone onboard when implementing falls screening	Clear instructions and action list	Clarify the first question
How to get everyone onboard when implementing falls screening	Needs to be incorporate into daily routines	Reminders
Applicable in many areas of health services but not in all	N/A	Annual health check-up
Applicable in many areas of health services but not in all	N/A	Communal activities

^a^ N/A = not applicable.

### Ethical Considerations

Ethical approval was obtained from the Ethical Review Authority (Reg. no. 2020–00134, 2021–04733, and 2022–02258-02). All participants who agreed to participate in the focus group interview or individual interview received both oral and written information about the purpose of the study and signed an informed consent form. They were informed that their participation was voluntary and that they could withdraw at any time. The study was conducted in accordance with the Helsinki Declaration.

### Role of the Funding Source

The funders have no role in design of the data collection, management, analysis, interpretation of data, writing manuscripts, or in the decision to submit for publication.

## RESULTS

**Figure 3:**
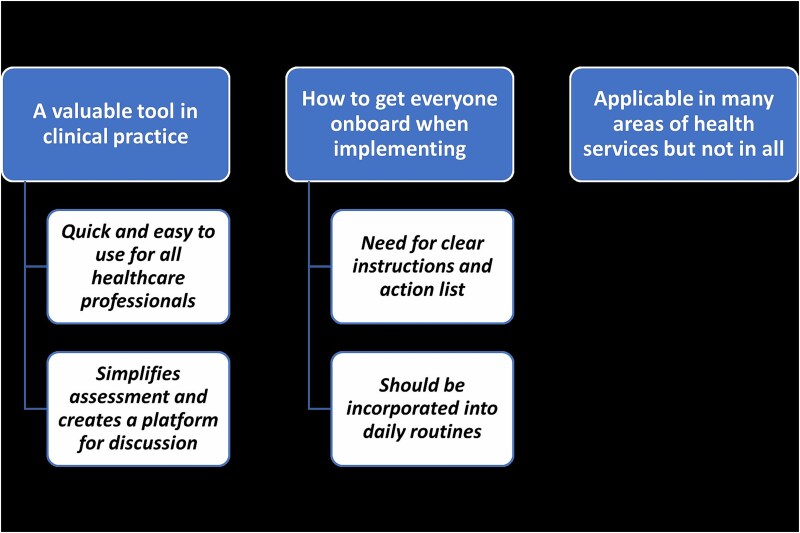
Themes and Subthemes.

The analysis resulted in 3 themes; “a valuable tool in clinical practice,” “how to get everyone onboard when implementing fall risk screening,” and “applicable in many areas of health services but not in all” with 4 related subthemes “quick and easy to use for all health care professionals,” “simplifies assessment and creates a platform for discussion,” “need for clear instructions and action list,” and “should be incorporate into daily routines” (Figure 3).

### A Valuable Tool in Clinical Practice

Participants experienced that the instrument worked well in a clinical context as it was perceived as clear and relevant. Participants described the FIF tool as an instrument easy to apply, both in a primary health care setting and in a public health project. The participants also described that it was important to work on fall prevention and that the FIF tool as a screening instrument could be valuable in preventing falls. FIF tool provided important information for further assessment efforts and facilitated efforts to successfully identify individuals who might otherwise had been missed.

#### Quick and Easy to Use for All Health Care Professionals

The participants expressed that the FIF tool was quick and easy to use, to familiarize themselves with, and to understand. Participants described that the questions were of the same character as in a standard anamnesis and therefore the questions did not feel strange, intrusive or unfamiliar to ask. The screening instrument was described by the participants as a short test and quicker to complete than expected which was perceived as an advantage due to time constrains in everyday work. The participants also described that it was easy to explain the results from the FIF tool to the person being screened. The performance of the balance test was also perceived as easy with clear instructions and easy to perform.

“Ah I can only agree. It was very easy to take, so it was quick to do. Nothing weird, just straight questions. Easy for the patients to answer. You didn’t have to explain the questions. And it was quick. And just like you said, it felt like the patients found it very smooth as well.” (Focus group interview 3).

#### Simplifies Assessment and Creates a Platform for Discussion

The participants described that screening for fall risk was valuable and important and a high priority in primary care. The participants described that primary prevention is important to enable health promotion. They also described that screening for fall risk opens up, creates an overview and raises awareness of the risks.

The participants described that screening was perceived as saving resources and could be tested on a larger group and used in public health work to provide the information on the need of health promotion interventions. The FIF tool was also found to be useful since it provided a basis for discussion with the person being tested and a motivation for interventions or reduce the risk of falling.

It was described by the participants that the balance test itself felt relevant as it was better to perform something concrete like standing on 1 leg instead of just talking about their fall risk. The balance test then became an eye-opener for the person being tested and provided important information about balance ability both for the participant and the person being screened. The participants perceived that the results from the balance test was easy to interpret. The participants described that the screening tool was a suitable test for fall prevention where people who were at risk of falling could be identified.

“The screening determines whether we should proceed or not. Because we might think that when they walk down the corridor, everything looks perfectly fine, or when we’re talking to them at home, there’s nothing indicating any risk of falling. And then, with a simple screening like this, we realize, ‘Ah, here we see something we could work on further.’ That’s really great!” (Individual interview 1).

“We’ve been able to choose whether they go to the falls prevention or promotion stage and there it’s actually been golden to use and easy for us to be able to separate.” (Focus group interview 1).

### How to Get Everyone Onboard When Implementing Fall Risk Screening

The participants experienced some ambiguity in items of the FIF tool and gave suggestions to facilitate and reduce the risk of misinterpretation of the instrument. It also became clear, from the participants, that the ability to use the screening instrument required organizational conditions. The participants described how the use of a new routine with a new screening instrument could be simplified and facilitated in the clinical reality.

#### Need for Clear Instructions and Action List

The participants described suggestions for improving the FIF tool. It mainly revolved around clarifying the FIF tool’s questions regarding IADL dependency, which could be interpreted in slightly different ways. The participants described that there was room for interpretation regarding the specific performance of the balance test, it could be interpreted differently depending on experience or profession. It occurred from the interviews that it was important that the instructions for the balance test were clear so that the person being tested could not compensate for their balance and thus be misclassified. It was suggested that an instructional video on how to perform the FIF tool should be made to further reduce the risk of misinterpretation.

“And then there’s one more thing I’ve been thinking about (balance test): how close to the wall one should stand or how to maintain visual contact with a fixed point and where that point should be. It can make a difference…” (Focus group interview 3).

The participants also described that a question could be added about the screened person’s perception of their balance ability as they felt that a person could get a low fall risk result but experience a fear of falling themselves. Participants described that the question about previous falls could be due to environmental factors and not just because of increased fall risk and therefore there is a risk of misinterpretation. The same question could also be difficult to answer for someone with cognitive impairment as it may be difficult to remember 3 years back in time. A suggestion was then that the time span could be shortened to 1 year instead. Another suggestion the participants had was to add an action list at the back of the instrument to make it easier to suggest interventions, especially for those participants who did not regularly work with fall risk assessments.

“And then there was the 1 about if you’ve fallen 3 years ago and then someone who had good balance had slipped on a patch of ice or something and broken something and been in hospital because they’ve slipped. So, it’s also a little bit of an interpretation question whether it’s because of impaired balance or just 1 because of the ice patch.” (Focus group interview 2).

#### Should be Incorporated Into Daily Routines

Implementing a new screening instrument requires different supportive and organizational conditions. The participants described that a new screening instrument needs to be easy to document for the health care professionals and integrated into the patient records/journal systems with standardized templates and keywords. A new routine needs to be established from the start so it could be incorporated in a daily routine. They also expressed a need for guidelines on which patient groups the instrument should be used on to facilitate routinization.

Participants described the importance to create a living culture of reminders and follow ups so that new routines are not forgotten and to facilitate a behavioral change in clinical practice. The participants also expressed that a new routine must be management driven and emphasized the importance of support and clarity from management.

The participants described the need to have a wide dissemination of knowledge in order to implement a new instrument and to reach out widely, for example at network meetings. There also needs to be a motivation from the management’s side explaining why to start using another tool.

“I believe that if we were to implement this in clinical practice, we should provide information about the background of the instrument and clarify that we have participated in this study as part of it. We should also emphasize its usability and simplicity, as well as how smoothly it operates. I think those are the key points.” (Individual interview 7).

Participants described that there may be those with difficulty dealing with changes, in these cases a transition period or grey zone can simplify a new routine where they allow some time to pass before they start using something new.

“Yes, but it’s probably good with such a transitional period. It can be. So that you bring along this last group that is still standing on the platform and hasn’t boarded this train. Who are at the front of the locomotive, who are in the middle, and who are still standing on the platform.” (Individual interview 7).

“Then you should go out with it broadly, it needs to be visible in different magazines, the physical therapy magazine and the like. It should be visible that there is this instrument and it is good for this.” (Individual interview 1).

### Applicable in Many Areas of Health Services But Not in All

Participants described for who and where the FIF tool could be used and where it might be appropriate for future implementation. Based on the participant’s different professions and backgrounds, several ideas for areas of future implementation were given, as well as where it might not be applicable to implement. The participants described different potential fields of use for the FIF tool, where the consensus was that it fit well into primary health care and public health settings.

In primary health care, it was suggested that all providers could use the FIF tool at new visits, mainly in the clinical setting. The participants suggested that FIF tool also could be used during the annual health check-up with the doctor or nurse at the health center. The participants expressed that it was important to reach older adults who were still healthy, therefore the FIF tool could be used on community-living older adults, identified through community activities or in public health settings. The participants described that the FIF tool could also be used in home help services to reach as many people as possible and to offer fall prevention interventions.

Home help services I definitely believe in. I think, I think home care could be something, I think there are a lot of people who have home help services but don’t have home health care rehabilitation. They can also screen and see that there is a problem and in that way you can also remove carpets, that you prevent it in some way.” (Individual interview 3).

On the other hand, the participants explored that it might not be perceived as relevant to use the FIF tool in home rehabilitation. Participants described that the primary purpose in home rehabilitation was to assess fall risk and therefore a screening tool was not as important in that context, and that they often meet frail older adults who already have a known fall risk.

“I think that, especially in home health care rehabilitation, it might happen automatically, where it is part of the first visit to go through the living environment and identify potential risks. Whereas, here, when we receive patients at the clinic, our focus is very much on what the patient is seeking help for, and there is very little time to address that specific issue. So, if you have a tool like this that screens quite quickly, it’s quite useful to use more. Because I think we’re generally a bit poor at using it unless they’re specifically seeking help for that particular problem.” (Focus group interview 3).

**Table 3:** i-PARIHS Core Elements Informing Interpretation of Participants Comments into Themes*^a^*

Core Elements	Characteristics
Innovation	Usability Clarity Degree of fit with existing practice and values Trialability
Recipients	Time Resources and support Existing networks Presence of boundaries Motivation and support
Context	Culture Learning environments Structure and system Organizational priorities Management support
Facilitation	Trialability Learning environment Management support Time and resources

^a^ i-PARIHS = integrated-Promoting Action on Research Implementation in Health Services.

## DISCUSSION

This interview study, which included both focus group and individual interviews, provides valuable insights on the use of the FIF tool in clinical practice and the potential barriers and facilitators that may arise during its implementation in a primary health care or public health setting using the i-PARIHS framework.^17^ The core elements characteristics for Innovation, Recipients, Context, and Facilitation are presented in Table 3.

### A Valuable Tool in Clinical Practice

They participants found the FIF tool to be an easy-to-use screening tool that worked well in both clinical practice and public health work. The health care professionals described the usability similarly to the older patients in our previous study.^18^ The tool’s performance was generally perceived as clear and relevant for its purpose. The participants noted that the FIF tool was quicker to complete than expected, which was seen as an advantage given the time, resources and support available in everyday work. The importance for a tool to be quick and easy to manage was highlighted in another study evaluating a brief-self assessment tool for cognitive impairment in primary care.^28^

They health care professionals also highlighted the benefits of early detection and identification with screening, such as giving support in an early stage of a disease to mitigate the development of the illness. Another study, investigating fall prevention routines in primary care, with interviews from different health care professionals, described that believing in the value of fall prevention was important for routinization of fall prevention in clinical practice.^29^ The health care professionals’ perception that screening is valuable could facilitate the implementation of the FIF tool.

### How to Get Everyone Onboard When Implementing Fall Risk Screening

The participants described that there was room for interpretation regarding the specific performance of the balance test, meaning it could be interpreted differently depending on experience or profession. The participants also emphasized the need for widespread dissemination of knowledge to effectively implement a new instrument. They highlighted the importance of reaching out broadly and extending existing networks.^17^

In another study when evaluating barriers and facilitators for implementation,^30^ the health care provider and clinical support staff highlighted that training and preparation was important for the implementation process. Yet another study described that it is important to give the health care professionals support and prepare them on how to use a new tool with their patients before the test period starts.^11^ In our study there were different health care professionals with different experience of working with fall prevention. That could be 1 reason why the FIF tool needs to be made clearer and include more information about the performance to reduce the risk of misinterpretation during the implementation process.

The interplay between a preventive approach and its context (eg, health care settings) can be highly intricate.^8^^.^ To accommodate the limited time available in everyday clinical practice, the participants emphasized that successful implementation of a new screening tool requires favorable organizational conditions. The key aspect of the FIF tool lies in its user-friendly design and seamless integration into patient records. This not only helps health care professionals in their daily tasks but also facilitates implementation of the tool. The participants highlighted the importance of creating a dynamic culture and learning environment with reminders and follow ups to facilitate a behavioral change in the clinical practice.^17^ Additional support from management is crucial to establish it as a standard practice.

The barriers for the implementation process were similar in a previous study, where the health care providers and clinical support staff identified factors such as increased workload, including time to conduct clinical assessments and increased documentation burden.^30^ On the other hand, more than half of the health care providers and clinical support staff in the study indicated that administrative leadership was neither a barrier nor a facilitator for the implementation support.

The participants described that there may be those with difficulty dealing with changes when implementing a new screening tool. Health care professionals described a need of motivation and support from the management, explaining why a new tool should be adopted. They stressed that new routines must be driven by the structure and systems, highlighting the importance of clarity and management support.^17^ The dissent to administratively imposed change was also a concern raised by health care providers, and it could be seen as another mandate that placed additional demands on their clinical time.^29^ The structure of the organizational systems in the primary health care where the FIF tool has been tested is an important factor in this context. The health care professionals indicated that using the screening instrument required organizational priorities, such as easy documentation in patient records with keywords or question text, and that it should be downloadable from the internet.^17^ To ensure alignment with existing practice and values, the health care providers recommended providing a brief summary of current clinical evidence, along with the necessary time, resources and management support to implement changes in clinical practise*.*^30^

### Applicable in Many Areas of Health Services But Not in All

The participants described different fields where the FIF tool could be used. The consensus was that it could be effectively implemented in many contexts, particularly in primary health care which increase the usability for the tool. However, the participants described presence of boundaries, noting that the primary purpose in home rehabilitation was already to assess fall risk, making a screening tool unnecessary.^17^ They describe that they often met frail older adults who already have a known fall risk and indicated that some older adults might have difficulty standing on 1 leg due to their need of a walker or wheelchair. In that case, the balance test could be time-consuming, and another screening tool could be more appropriate. Therefore, a screening tool was not as important for implementation in that context.

### Strengths and Limitations

When assessing the trustworthiness of our study we used credibility, dependability, confirmability and transferability.^27^ One strength in terms of credibility was the detailed description of the analytical process and that, in addition to the first author, 2 other researchers were also involved in the process of analyzing the data material (P.K., A-K.W.).

Regarding dependability, interviews were conducted until both the first and the second author experienced saturation.^26^ Saturation was reached when the aim of identifying barriers and facilitators based on the participants’ experiences of using the FIF tool were not seen to change the codes, subthemes or final themes from the data material.^26^According to confirmability, both the first and the second author are physical therapists and were aware that their pre-understanding of both the clinical environment and the subject affected their analysis of the data material.

The choice of 3 primary health care rehabilitation clinics and 2 primary health care centers within the Stockholm region and 1 public health project in Skåne region increases the study’s transferability. It also gave us a variety of health care professionals of using the FIF tool in different settings, which in turn may have provided a broader picture of the barriers and facilitators for implementing the screening tool.

One weakness could be the first 2 authors’ subjectivity and pre-understanding, as they were both familiar with the participant discussed during the interviews. It was therefore particularly important for additional authors to be involved during the analysis process. Regardless of whether the authors who carried out the data analysis attempted to be objective and compared their interpretations, the degree of subjectivity will affect their interpretation of the analysis process. A further weakness of the study could be that the health care professionals received different kinds of introduction to the FIF tool with orally or written instructions depending on the practical possibility or specific wishes from the health care professionals.

## CONCLUSION AND IMPLICATIONS

FIF tool appears to be a valuable screening tool for predicting first-time injurious falls in older adults, suitable for use by various health care professionals. Participants expressed that the FIF tool worked well in primary health care and public health settings; it was easy to use and meaningful for preventing falls. However, there was some room for improvements enhance clarity and mitigate potential misinterpretations. To facilitate successful implementation using the i-PARIHS framework, understanding the barriers and facilitators identified by health care professionals is crucial.^17^ The instrument should be easily accessible, straightforward to document in medical records and supported by management to establish it as a new routine, especially given the limited time available in everyday clinical practice. This knowledge informs the trialability that the instrument needs to be facilitated with a structure and system included a learning environment with management support, time and resources. However, this indicated also that the trialability could be more difficult in settings where these facilitators are not applicable.

## Supplementary Material

2024-0173_R1_Supplementary_Material_pzaf018

## Data Availability

The data in the study are pseudonymized (coded) personal data, and European General Data Protection Regulation prohibits us from sharing this completely open. The dataset only includes data from 20 human research participants. Due to the small sample size, there is a risk of identification of individual participants even though the data is de-identified. Data is available upon request, and requests for access to the data can be put to our Research Data Office (rdo@ki.se) at Karolinska Institutet.
