# Supplementary material for: Health Care Professionals’ Views of Barriers and Facilitators for Implementing a Fall Risk Screening Tool in Clinical and Public Health Settings
Source: Phys Ther. 2025 Feb 18;105(4):pzaf018. doi: 10.1093/ptj/pzaf018 (PMC12017392; doi:10.1093/ptj/pzaf018)
Supplement: 2024-0173_R1_Supplementary_Material_pzaf018 [file 2024-0173_r1_supplementary_material_pzaf018.pdf]

Supplementary Material 1. First-time Injurious Falls -FIF screening tool.

Have you ever had to seek immediate care because of a fall in the last three years?	Yes	□	No	□

(***No?*** Please answer the following questions)

			Women	Men
How old are you?	60–69	□	0	0
	70–79	□	1	2
	80–89	□	2	3
	90+	□	4	4
Do you live with someone?	Yes	□	0	0
	No	□	1	1
Do you need help in any or several of the following: • managing financies • using telephone • grocery shopping • using public transportation • preparing meals • cleaning • doing laundry	Yes	□	2	1
	No	□	0	0
**Physical test** (eyes open - 2 attempts/leg – best attempts counts)				
One-leg standing	<5 seconds	□	1	1
	≥5 seconds	□	0	0
	Total score =		___ **/8**	___ **/7**

**Low fall risk**	**High fall risk**
**No previous falls**	**Previous falls**
**0-2 points**	**3+ points**

Supplementary Material 2. Semi-structured interview guide for healthcare professionals

Description of the background to the study.

1. What do you associate with screening?
2. What do you think about when I say injurious falls?
3. How did you find testing the FIF tool with your patients?
4. Did you experience any difficulties using the FIF-tool and if so what cause the difficulties?
5. Did you feel comfortable asking the questions included in the FIF tool? Why/why not?
6. Were there any questions that were unclear in the FIF tool?
7. Were there any questions that was hard trying to explain to your patients?
8. How did you find performing the balance test in the FIF tool?
9. Did you find the instructions of the balance test to be clear enough?
10. What do you associate when I say “screening”?
11. Screening is done as a first step when trying to select patients who might need a more thorough assessment regarding fall risk. What meaning does screening have to the primary healthcare?
12. What did you feel when handing out the results from the FIF tool?
13. Do you feel that you have an idea about further assessment if your patient has a potential high fall risk?
14. Do you feel that FIF tool need any improvements and if so what kind of improvements?
15. How do you think that we could use FIF tool in an optimal way in primary healthcare?
16. What do you think is important to keep in mind for the continued use of the FIF tool?
17. What are the facilitators for you to continue using FIF tool?

18. Do you see any obstacles to be continued to use the FIF tool?
19. What were your expectations from FIF tool and if so, did FIF tool meet your expectations? For example, home rehabilitation or those seeking care here at the clinic?
20. Do you have any thoughts on which patient groups the FIF tool is useful for?
21. Do you have any other thoughts or comments that you would like to mention?

Additional questions for managers:

1. What conditions exist for you to continue using the FIF tool in primary healthcare from an organizational perspective?
2. Do you see any obstacles to the continued use of the FIF tool in primary healthcare from your perspective as a manager?
3. What do you think is important to consider from an organizational perspective as a manager when implementing the FIF tool?
